# Risk Factors of Distant Recurrence and Dissemination of *IDH* Wild-Type Glioblastoma: A Single-Center Study and Meta-Analysis

**DOI:** 10.3390/cancers16162873

**Published:** 2024-08-18

**Authors:** Takahiro Tsuchiya, Daisuke Kawauchi, Makoto Ohno, Yasuji Miyakita, Masamichi Takahashi, Shunsuke Yanagisawa, Sho Osawa, Shohei Fujita, Takaki Omura, Yoshitaka Narita

**Affiliations:** Department of Neurosurgery and Neuro-Oncology, National Cancer Center Hospital, Tokyo 104-0045, Japan; mephymach@gmail.com (T.T.); dakawau2@ncc.go.jp (D.K.); mohno@ncc.go.jp (M.O.); yasuji.miyakita@jfcr.or.jp (Y.M.); masataka@tokai.ac.jp (M.T.); shuyanag@ncc.go.jp (S.Y.); soosawa@ncc.go.jp (S.O.); s8o59.so3@gmail.com (S.F.); tomura@ncc.go.jp (T.O.)

**Keywords:** glioblastoma, recurrence, dissemination, subventricular zone, lateral ventricle, tumor location, ventricular opening, ventricular entry

## Abstract

**Simple Summary:**

This study identified the risk factors of non-local recurrence of isocitrate dehydrogenase (*IDH*) wild-type glioblastoma, a highly aggressive brain tumor. We found that subventricular zone involvement significantly increased the risk of non-local recurrence, with the tumor contacting the trigone of the lateral ventricle and tending to develop subependymal dissemination. A meta-analysis of previous studies confirmed subventricular zone involvement and O-6-methylguanine DNA methyltransferase promoter methylation as risk factors for non-local recurrence. In contrast, ventricular opening via surgery did not increase the risk of non-local recurrence. These findings emphasize the need for tailored therapeutic strategies based on tumor location and molecular characteristics and provide valuable guidance for clinical decision-making in managing recurrent glioblastoma.

**Abstract:**

Isocitrate dehydrogenase (*IDH*) wild-type glioblastoma (GBM) is a highly aggressive brain tumor with a high recurrence rate despite adjuvant treatment. This study aimed to evaluate the risk factors for non-local recurrence of GBM. In the present study, we analyzed 104 GBMs with a single lesion (non-multifocal or multicentric). Univariate analysis revealed that subventricular zone (SVZ) involvement was significantly associated with non-local recurrence (hazard ratio [HR]: 2.09 [1.08–4.05]). Tumors in contact with the trigone of the lateral ventricle tended to develop subependymal dissemination (*p* = 0.008). Ventricular opening via surgery did not increase the risk of non-local recurrence in patients with SVZ involvement (*p* = 0.190). A systematic review was performed to investigate the risk of non-local recurrence, and 21 studies were identified. A meta-analysis of previous studies confirmed SVZ involvement (odds ratio [OR]: 1.30 [1.01–1.67]) and *O*-6-methylguanine DNA methyltransferase promoter methylation (OR: 1.55 [1.09–2.20]) as significant risk factors for local recurrence. A time-dependent meta-analysis revealed a significant association between SVZ involvement and dissemination (HR: 1.69 [1.09–2.63]), while no significant association was found for distant recurrence (HR: 1.29 [0.74–2.27]). Understanding SVZ involvement and specific tumor locations associated with non-local recurrence provides critical insights for the management of GBM.

## 1. Introduction

Isocitrate dehydrogenase (*IDH*) wild-type glioblastoma (GBM) is the most common and aggressive primary brain tumor in adults [1]. Most patients demonstrate recurrence even after postoperative radiotherapy (RT) plus temozolomide (TMZ). GBM recurrence patterns include local and non-local recurrences. The most predominant initial recurrence is local; in 78–95% of cases, the tumor manifests within 2–3 cm of the resection cavity [2,3,4]. During the later stages of the disease, GBMs tend to recur in parenchyma away from the initial tumor or spread in the cerebrospinal fluid (CSF) [5,6,7]. The prognosis is unfavorable, especially for non-local recurrence, with a median overall survival of 3.5 months from the time of leptomeningeal dissemination and a median overall survival of less than 2 months with the best supportive care alone [8,9]. Understanding the risk factors and clinical characteristics of non-local recurrence can help make decisions for patients with end-stage GBM [10]. Previous studies of GBM recurrence patterns have examined the association between various risk factors and non-local recurrence. These include the location of the GBM, ventricular opening due to a surgical procedure, and *O*-6-methylguanine DNA methyltransferase (*MGMT*) promoter methylation status [11,12,13,14,15,16,17,18,19,20,21,22,23]. However, the results of these studies are conflicting, and no definitive consensus has been reached. Non-local recurrence includes distant parenchymal recurrence and CSF dissemination; however, there is no consensus on the definitions of these terms or how they are determined. Therefore, the purpose of this single-center, retrospective cohort study was to comprehensively examine the risk of non-local recurrence according to the location of GBM and other factors. Furthermore, by reviewing the previous literature, we aimed to synthesize the results of previous studies and provide current knowledge regarding the devastating condition of non-local recurrence in GBM.

## 2. Materials and Methods

### 2.1. Patient Selection

This study included patients with newly diagnosed *IDH* wild-type GBM with a Karnofsky performance status score (KPS) of 70 or more who were treated at the National Cancer Center, Tokyo, between January 2015 and December 2019. Patients who experienced recurrence were excluded from the study. All patients underwent postoperative RT with concomitant and adjuvant TMZ therapy. Of these patients, 104 consecutive cases with a single lesion (non-multifocal or multicentric) were enrolled in this study. The remaining 16 patients were excluded from the study for the following reasons: 14 patients had multifocal tumors and/or dissemination before the initial surgery, and 2 patients had no preoperative images available for review. These patient selection criteria were confirmed by two independent authors (T.T. and D.K.). All patients were diagnosed with *IDH* wild-type GBM based on neuropathological examination of tumor specimens according to the 5th edition of the WHO classification [24]. Patients who were initially treated elsewhere and referred to our hospital for further treatment at the time of recurrence were eligible for enrollment if their clinical course and radiological information were available.

The clinical information, operative records, and radiological information of the patients were reviewed. The following data were collected for each patient: age at diagnosis, sex, preoperative KPS, tumor molecular status (*IDH*1/2 mutation, *TERT* promoter mutation, and *MGMT* promoter methylation), tumor location, maximum tumor size, tumor proximity to the ventricle, ventricular opening by operation, extent of resection, postoperative ischemia, postoperative initial adjuvant therapy, date of the tumor recurrence, and date of death or last hospital visit.

Tumor proximity to the ventricle was determined using preoperative magnetic resonance imaging (MRI). The tumor was classified to be in contact with the subventricular zone (SVZ) if the tumor postcontrast enhancement adjoined the lateral ventricular ependyma (≥5 mm). Subsequently, the region where the tumor was in contact with the SVZ was classified as the anterior horn, body, trigone, or inferior horn of the lateral ventricle. Operative ventricular opening was determined based on the surgeon’s operative notes and postoperative imaging studies. The extents of resection were gross total resection (GTR; 100%), subtotal resection (STR; 95–99%), and otherwise partial resection (PR) by assessing residual tumor enhancement on MRI within 48 h after surgery. Postoperative ischemia was determined by the presence of a postoperative high diffusion-weighted imaging (DWI) signal around the extraction cavity.

This study was conducted in accordance with the Declaration of Helsinki, and the protocol was approved by the Internal Review Board of the National Cancer Center (Research Project number: 2013-042). All participants gave their informed consent for inclusion before they participated in this study.

### 2.2. Recurrence Definition

The patients were evaluated using contrast-enhanced T1- and T2-weighted imaging or fluid-attenuated inversion recovery (FLAIR) before and after surgery. They were followed up using MRI at the time of and one month after the completion of RT and every two months thereafter or according to clinical symptoms.

**Figure 1:**
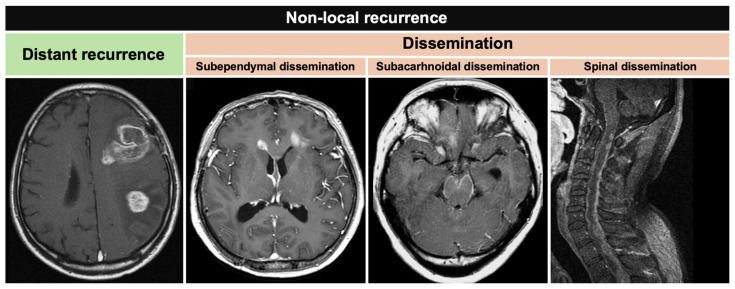
Definition of non-local recurrence. Distant recurrence (intraparenchymal, new contrast-enhancing foci that were noncontiguous with the resection cavity or remnants of the original) and dissemination (distant from the original tumor and exposed to the CSF space) were classified as non-local recurrence. Dissemination included subependymal, subarachnoidal, and spinal.

The patterns of recurrence were as follows: local recurrence (at the regional tumor), distant recurrence (intraparenchymal, new contrast-enhancing foci contiguous with the resection cavity or remnants of the original tumor), and dissemination (distant from the original tumor and exposed to the CSF space). Distant recurrence and dissemination were classified as non-local recurrence (Figure 1). Dissemination was subependymal, subarachnoidal, or spinal. When pseudo-progression was suspected, positron emission tomography or surgical procedures were performed for differential diagnosis, as necessary. The patients were observed without changing the adjuvant chemotherapy if the lesions were stable. Pseudo-progression represented the resolution of lesions during follow-up.

Tumors diagnosed as local at the first recurrence were followed up until the second progression, and the patterns of recurrence were evaluated. Tumors diagnosed as local at the second recurrence were followed up until the third progression, and the patterns of recurrence were evaluated. Cases of patient deterioration without radiological tumor progression were classified as clinically progressive disease (clinical PD). Cases with no tumor progression, with the patient alive at the time of the final follow-up, were classified as alive without recurrence. Cases with no tumor progression at the time of the final follow-up but with the patient lost to follow-up were classified as lost.

The time to local recurrence was calculated from the day of the initial surgery to the day of local recurrence. Progression-free survival (PFS) was calculated from the day of initial surgery to any type of recurrence or death from any cause. However, for cases of distant or disseminated recurrence, death and loss to follow-up were censored on the day of each event. The time to distant recurrence was calculated from the day of the initial surgery to the day of distant recurrence. Disseminated patterns of recurrence, death, and loss to follow-up were defined as those that were censored on the day of each event. The time to disseminated recurrence was calculated from the day of the initial surgery to the day of disseminated recurrence. Distant patterns of recurrence, death, and loss to follow-up were censored on the day of each event. Patients alive without any type of recurrence at the final follow-up were censored on the day of the final follow-up.

### 2.3. Molecular Analysis

DNA was extracted from frozen tumor tissues using a DNeasy Blood & Tissue Kit (Qiagen, Tokyo, Japan). The presence of hotspot mutations in *IDH1* (R132) and *IDH2* (R172) was assessed by pyrosequencing, as previously described, which was designed to detect all known mutations in these codons [25]. The methylation status of the *MGMT* promoter was analyzed using the bisulfite modification of tumor genomic DNA followed by pyrosequencing, as previously described [26]. Methylation was defined as positive when the mean level of methylation at the 16 CpG sites examined was greater than 16% [26,27]. One patient was excluded from the analysis due to insufficient sample quality for methylation data acquisition.

### 2.4. Systematic Review of the Literature

A systematic literature review was conducted in June of 2024 using the Preferred Reporting Items for Systematic Reviews and Meta-Analyses (PRISMA) guidelines [28]. The search was conducted in PubMed for full-text articles reporting the risk factors for non-local recurrence, including distant and disseminated recurrence, in patients with *IDH* wild-type GBM. The search strategy consisted of the terms “glioblastoma”, “distant”, “dissemination”, “recurrence”, and/or “relapse”. Review articles, studies that included non-GBM or recurrent GBM, and non-English-language publications were excluded. Eligibility screening and data extraction from titles, abstracts, and subsequent full-text articles were performed by two independent reviewers (T.T. and M.O.). Only articles in which odds ratios (OR) or hazard ratios (HR) were provided in the text or from which they could be calculated were included in the meta-analysis. The *I*^2^ statistic was used to assess the heterogeneity of the studies.

### 2.5. Statistical Analysis

Continuous variables were presented as medians and interquartile ranges (IQR). Categorical variables were presented as absolute numbers and percentages. The patient overall survival (OS) and PFS were calculated using the Kaplan-Meier method and presented as medians with 95% confidence intervals (CI). The significance of the differences in nominal variables was evaluated using Fisher’s exact test. The durations from the initial surgery to non-local recurrence, distant recurrence, and dissemination were calculated using the Kaplan-Meier method and compared using the log-rank test. Potential prognostic factors were initially evaluated using a univariate Cox proportional hazards model to assess the risk factors of non-local recurrence. Variables with *p*-values of <0.200 were subjected to further evaluation using a multivariable Cox proportional hazard model. The results of the Cox analyses are presented as HR with 95% CI and a *p*-value. The statistical analyses were conducted using R software (version 4.0.2; The R Project for Statistical Computing, Vienna, Austria), and statistical significance was set at a *p*-value less than 0.05.

## 3. Results

### 3.1. Clinical Characteristics

**Figure 2:**
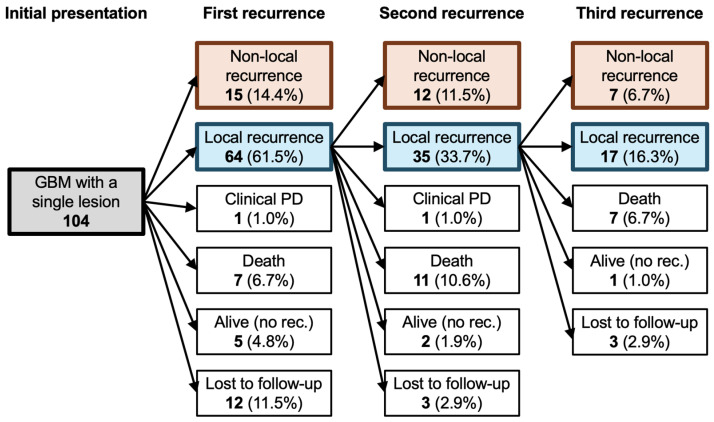
The tree diagram of the pattern of GBM recurrence. Of 104 patients, 79 (76.0%) developed first recurrent diseases: 64 (81.0%) were local recurrences and 15 (19.0%) were non-local recurrences. Of 64 patients with local recurrence at the first recurrence, 47 (73.4%) developed second recurrences: 35 (74.5%) were local recurrences, and 12 (25.5%) were non-local recurrences. Of 35 patients with local recurrence at the second recurrence, 24 (68.6%) developed third recurrences: 17 (70.8%) were local recurrences, and 7 (29.2%) were non-local recurrences. PD: progressive disease.

The clinical characteristics of the patients are presented in Table 1. The median age at diagnosis was 61.5 years, and 66 patients (63.5%) were male. The median KPS score at admission was 80. There were 40 patients (38.5%) who exhibited *MGMT* promoter methylation, whereas *TERT* promoter mutations were identified in 69 patients (66.3%), with C228T and C250T mutations in 52 (50.0%) and 17 (16.3%) patients, respectively. The tumor locations were as follows: 37 (35.6%), 31 (29.8%), 18 (17.3%), 7 (6.7%), and 4 (3.8%) patients had tumors in the frontal lobe, parietal lobe, temporal lobe, spread in two or three lobes, and infratentorial region, respectively. There were 54 (51.9%) tumors that were left-sided, 46 (44.2%) were right-sided, and 4 (3.8%) were on the midline. The median maximum tumor size was 39.5 mm. SVZ involvement was observed in 57 patients (54.8%). The initial postoperative adjuvant therapies included RT with TMZ for 45 patients (43.3%), RT with TMZ plus bevacizumab (BEV) for 20 patients (19.2%), and RT with TMZ plus Gliadel wafer for 6 patients (5.8%). The median follow-up duration was 24.8 months. Ventricular opening by operation was performed for 30 patients (28.8%). The extent of resection was GTR for 37 patients (35.6%), STR for 21 patients (20.2%), PR for 29 patients (27.9%), and biopsy for 17 patients (16.3%). There were 24 patients (23.1%) who showed ischemia around the extraction cavity on postoperative MRI. The median OS, PFS, and non-local, recurrence-free survival were 32.1 months, 9.6 months, and 41.6 months, respectively. Distant recurrence was observed in 13 patients (12.3%), and dissemination was observed in 25 patients (24.0%). Of 104 patients, 79 (76.0%) developed recurrent disease: 64 (81.0%) and 15 (19.0%) had local and non-local recurrence, respectively. Of 64 patients with local recurrence as the first recurrence, 47 (73.4%) developed a second recurrence, 35 (74.5%) developed local recurrence, and 12 (25.5%) developed non-local recurrence. Of 35 patients with local recurrence as the second recurrence, 24 (68.6%) developed a third recurrence, 17 (70.8%) had local recurrence, and 7 (29.2%) had non-local recurrence. A tree diagram illustrates the pattern of recurrence for up to the third time (Figure 2). Of 104 patients, 41 patients (39.4%) were alive without recurrence, and 8 patients (7.7%) were dead and 11 patients (17.3%) were lost to follow-up for palliative care at 1 year. Local recurrence was 45 patients (43.3%), distant recurrence was 2 patients (1.9%), and dissemination was 5 patients (4.8%) at 1 year. At 2 years after surgery, 15 patients (14.4%) were alive without recurrence, 29 patients (27.9%) were dead, and 18 patients (17.3%) were lost to follow-up, shown in the graphical abstract. Local recurrence was 51 patients (total 49.1%, alive 30.8%, dead 15.4%, lost to follow-up 2.9%), distant recurrence was 7 patients (total 6.7%, alive 3.8%, dead 1.9%, lost to follow-up 1.0%), and dissemination was 15 patients (total 14.4%, alive 5.8%, dead 6.7%, lost to follow-up 1.9%).

### 3.2. Risk Factors of Non-Local Recurrence

To determine the risk factors for the development of distant recurrence or dissemination (non-local recurrence), we initially conducted a univariate analysis of the data of 104 patients using the following clinical and genetic variables: age, sex, KPS score on admission, *MGMT* promoter methylation, *TERT* promoter mutation, tumor location, maximum tumor size, SVZ involvement, ventricular opening, extent of resection, and postoperative ischemia. Univariate analysis demonstrated that SVZ involvement and ventricular opening were significantly associated with the development of non-local recurrence (Table 2). Subsequently, multivariate analyses were performed using the following variables: KPS on admission, MGMT promoter methylation, SVZ involvement, ventricular opening, extent of resection, and postoperative ischemia. The results of multivariate analysis indicated that these factors were not significantly associated with non-local recurrence.

### 3.3. Patterns of Non-Local Recurrence According to Tumor Location

**Figure 3:**
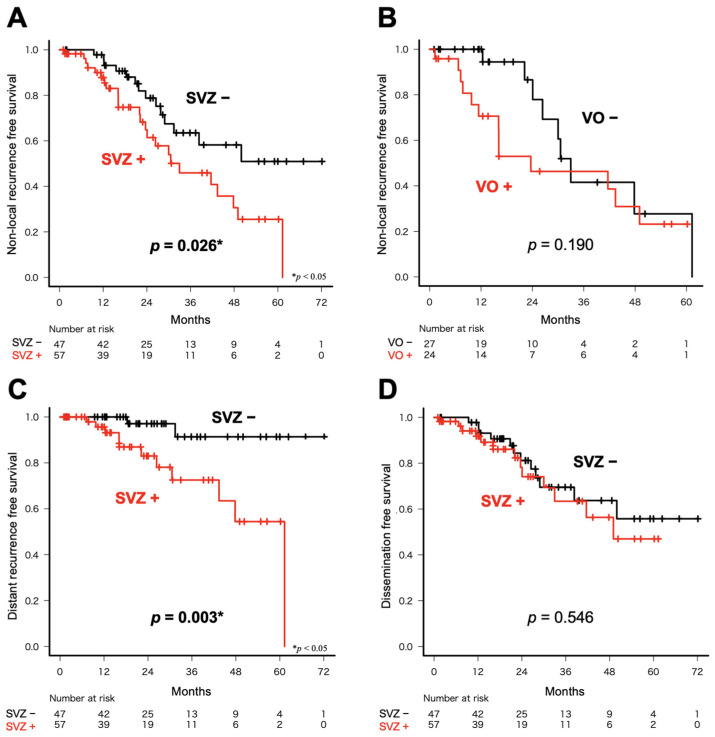
(**A**) A significant difference was observed between the times to non-local recurrence for the patients with and without SVZ involvement (*p* = 0.026). (**B**) An analysis of 51 patients with SVZ lesions for whom information on the presence of ventricular opening was available demonstrated no significant differences in the presence of ventricular opening and time to non-local recurrence (*p* = 0.190). (**C**) SVZ involvement was significantly associated with early distant recurrence (*p* = 0.003). (**D**) There were no significant differences in the SVZ involvement and time to dissemination (*p* = 0.546). Death and loss to follow-up were censored in this analysis. VO: ventricular opening.

The preceding analysis indicated that SVZ involvement and ventricular opening affected the patterns of non-local recurrence. Subsequently, the characteristics of non-local recurrence of 104 *IDH* wild-type GBMs were analyzed, with a focus on SVZ involvement and location. A significant difference was observed in the time to non-local recurrence in patients with and without SVZ involvement (Figure 3A, *p* = 0.026). In contrast, an analysis of 51 patients with SVZ involvement for whom information on the presence of ventricular opening was available demonstrated no significant difference between the presence of ventricular opening and the time to non-local recurrence (Figure 3B, *p* = 0.190). Of the cases of non-local recurrence, distant metastasis and dissemination were examined, and SVZ involvement was significantly associated with early distant recurrence (Figure 3C, *p* = 0.003); however, there was no significant difference between SVZ involvement and time to dissemination (Figure 3D, *p* = 0.546).

**Figure 4:**
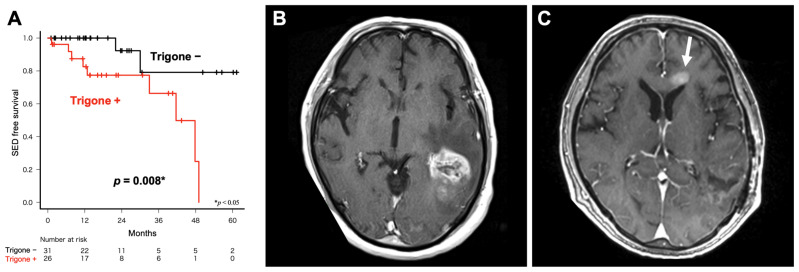
Relationship between the trigone of the lateral ventricle and subependymal dissemination. (**A**) Trigone group showed significantly earlier subependymal dissemination (*p* = 0.190, SED: subependymal dissemination). (**B**) Representative MRI of *IDH* wild-type GBM in contact with the trigone of the lateral ventricle at the initial onset. (**C**) Non-local recurrence with subependymal dissemination (arrow) in the same patient.

A total of 57 *IDH* wild-type GBMs with SVZ involvement, including specific types of non-local recurrence, were analyzed in detail. SVZ involvement was classified according to the site of involvement: the anterior horn, body, trigone, or inferior horn of the lateral ventricle (Table 3). The anterior and inferior horn groups had the highest incidence of distant recurrence (33.3% and 40.0%, respectively), whereas the body group demonstrated no non-local recurrence. The trigone group had the highest incidence of subependymal dissemination (35.6%). The trigone group demonstrated a significant risk of progression to subependymal dissemination (OR: 7.405, 95% CI: 1.315–78.237, *p* = 0.016), as well as a significant risk of early subependymal dissemination (Figure 4A, *p* = 0.008). Representative MRI images of *IDH* wild-type GBM in contact with the trigone of the lateral ventricle at initial onset (Figure 4B) and non-local recurrence with subependymal dissemination in the same patient are shown (Figure 4C).

### 3.4. Systematic Literature Review and Meta-Analysis

**Figure 5:**
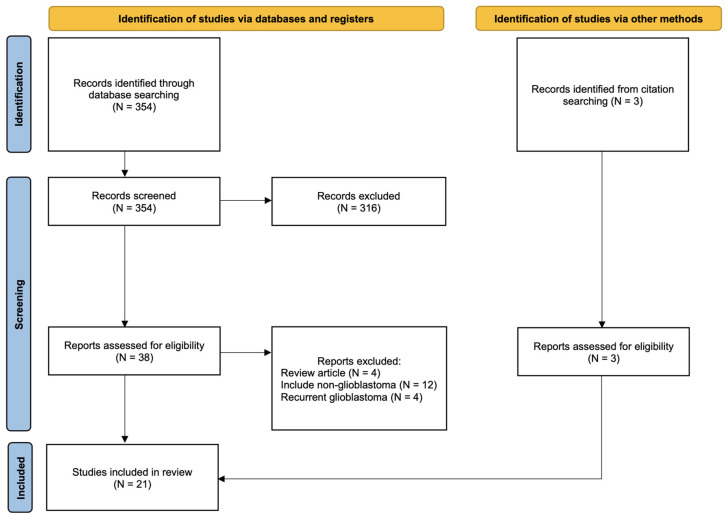
Flowchart of the study selection process.

A total of 354 studies were identified during the initial literature search. There were 2 studies identified through a review of the study references, resulting in 356 screened studies. A total of 316 of the 356 studies were excluded due to irrelevance of their titles or abstracts to the current study or publication in a non-English language. A total of 40 full-text articles were obtained and reviewed. Of those, 20 studies were excluded for several reasons, including review articles and studies that included patients with non-GBMs or recurrent GBM. A total of 21 studies were identified and included in the analysis in addition to ours. A flowchart of the study selection process is shown in Figure 5.

A summary of previous studies on the incidence of and risk factors for the non-local recurrence of GBM is presented in Table 4 [5,11,12,13,14,15,16,17,18,19,20,21,22,23,29,30,31,32,33,34,35]. The median sample size of these previous studies was 142 (range 49–607), and the incidence of non-local recurrence was 13.3–55.1%. As risk factors for non-local recurrence, SVZ involvement, ventricular opening, and *MGMT* methylation were examined in seven, four, and five studies, respectively. Other risk factors include BEV use and postoperative ischemia.

**Figure 6:**
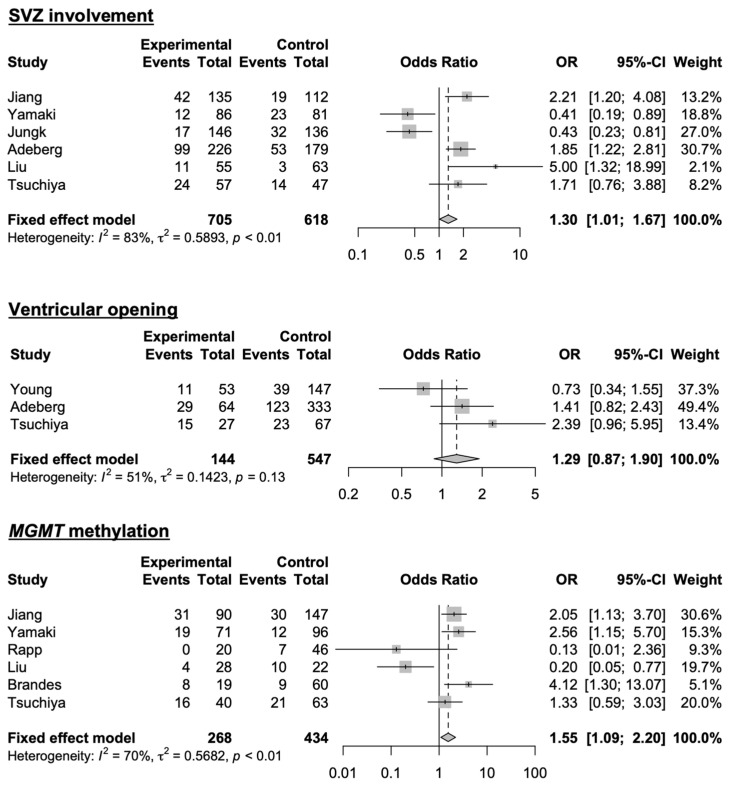
Forest plots of studies evaluating the risk of SVZ involvement, ventricular opening, and *MGMT* methylation for non-local recurrence [5,11,12,13,14,16,17,20].

**Figure 7:**
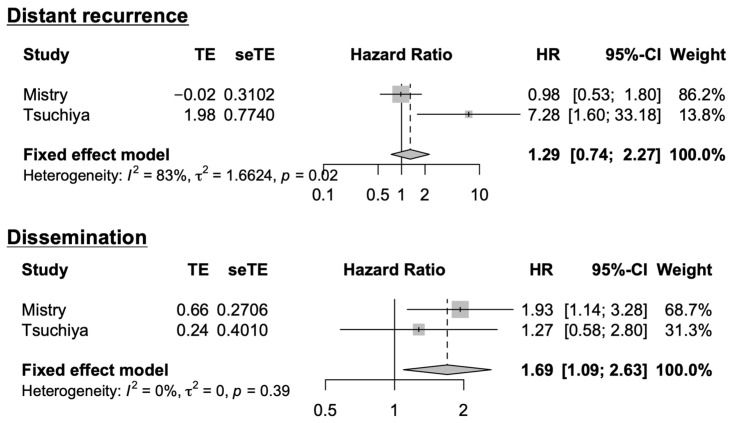
Forest plots of studies evaluating the risk of SVZ involvement for distant recurrence and dissemination [15].

A meta-analysis was conducted to integrate our study with previous studies in which the ORs for non-local recurrence were available for SVZ involvement, ventricular opening, and *MGMT* methylation (Figure 6). A total of three studies, including ours, were included in the meta-analysis for SVZ involvement: The results indicated a significant association between non-local recurrence and SVZ involvement (OR: 1.30, 95% CI: 1.01–1.67). A total of three studies, including ours, were included in the meta-analysis for ventricular opening, and the results indicated no significant association between non-local recurrence and ventricular opening (OR: 1.29; 95% CI: 0.87–1.90). A total of three studies, including ours, were included in the meta-analysis for *MGMT* methylation status: The results indicated a significant association between non-local recurrence and *MGMT* methylation status (OR: 1.55, 95% CI: 1.09–2.20). Among these meta-analyses, there was a relatively high inter-study heterogeneity for SVZ involvement and *MGMT* methylation (*I*^2^ = 83%, 70%), whereas no significant heterogeneity was observed for ventricular opening (*I*^2^ = 51%). Only two studies, including ours, presented HRs for SVZ involvement for the risk of distant recurrence and dissemination (Figure 7). A meta-analysis of these studies revealed no significant association between SVZ involvement and distant recurrence (HR: 1.29; 95% CI: 0.74–2.27). On the other hand, there was a significant association between SVZ involvement and dissemination (HR: 1.69; 95% CI: 1.09–2.63). There was considerable heterogeneity between the studies on SVZ involvement and distant recurrence (*I*^2^ = 83%), whereas no significant heterogeneity was observed for SVZ involvement and dissemination (*I*^2^ = 0%).

## 4. Discussion

This study comprehensively reviewed and clarified the risk factors for non-local recurrence of *IDH* in wild-type GBM. A systematic review of previous literature was conducted, and the observations were integrated into the meta-analysis to resolve the discrepancies among previous studies. Additionally, a time-dependent analysis of the risk of non-local recurrence of *IDH* wild-type GBM was conducted. To date, only two studies have been conducted, including the present study. In addition to demonstrating that SVZ involvement is associated with non-local recurrence, this is the first study to suggest that GBM in contact with the trigone of the lateral ventricle may be associated with the development of subependymal dissemination.

In this study, non-local recurrence was defined as distant recurrence and dissemination. However, previous studies have often used inconsistent or ambiguous definitions for these terms. Some studies have referred to non-local recurrence as “distant recurrence”, while others have classified it as distant recurrence and dissemination, as we did. The diagnosis of dissemination is primarily based on MRI findings, and it is impractical to perform a lumbar puncture for all cases. Therefore, pathologically proven cases are limited. Moreover, MRI is rarely performed shortly before death in patients with end-stage GBM, especially those receiving the best supportive care (BSC), making it challenging to ascertain the precise incidence of non-local recurrence at the time of death. Therefore, our systematic review indicated that the incidence of non-local recurrence in *IDH* wild-type GBM is 13.3–55.1%, but this figure may be underestimated. In contrast, Inoue et al. demonstrated that preoperative leptomeningeal enhancement does not necessarily indicate untreatable leptomeningeal dissemination [36]. Autopsy of patients with GBM is the most accurate method to confirm the presence of non-local recurrence. However, ethical considerations and a limited number of reports on this topic present challenges. Onda et al. performed complete autopsies of 51 patients who died of cerebral glioblastoma, and 14 had dissemination into the CSF [37]. However, the mechanisms underlying distant recurrence remain unclear. One hypothesis suggests that GBM cells may invade white matter tracts or migrate and spread throughout the SVZ [29,38,39,40], while another hypothesis proposes that they may spread via the CSF [20,41,42]. Our results for 104 patients with *IDH* wild-type GBM indicated a high incidence of distant recurrence in patients with SVZ involvement, supporting the hypothesis that this occurs via subependymal spread or the CSF. We previously reported that infratentorial dissemination occurs via this pathway. [43]

In the meta-analysis, SVZ involvement was associated with non-local recurrence; however, there was heterogeneity among the studies. Yamaki et al. and Jungk et al. concluded that SVZ involvement was a negative risk factor for non-local recurrence [14,16], which is inconsistent with the results of our study and meta-analysis. This discrepancy may be attributed to differences in the definitions of non-local recurrence, surgical removal strategies, extent of resection, and type of treatment for recurrence. With regard to the definition of non-local recurrence, some studies defined the recurrence outside the irradiated field as non-local recurrence, some defined it by distance from the primary lesion, and some did not specify the definition of non-local recurrence. Therefore, our intended local recurrence may be potentially included in the non-local recurrence in the previous studies and vice versa. SVZ is an important niche for neural and glioblastoma stem cells (GSCs) [44,45]. It is associated with resistance to radiotherapy and chemotherapy and recurrence [46,47]. We further classified SVZ locations and examined their associations with the types of non-local recurrence for the first time. Our findings reveal a significant association between contact with the trigone of the lateral ventricle and subependymal dissemination. Distant recurrences were observed to occur with greater frequency in the anterior and inferior horns of the lateral ventricle and with lesser frequency in the trigone of the lateral ventricle. These results suggest the presence of specific anatomical features in the SVZ of GSCs. In other words, the results indicate that the SVZ of the trigone of the lateral ventricle has characteristics that render it susceptible to subependymal dissemination in non-local recurrences. Thus, the clinical implication is that MRI should be monitored with particular attention to the presence of subependymal dissemination during the follow-up of patients with GBM occurring in contact with the trigone of the lateral ventricle. The involvement of the SVZ was evaluated using contrast-enhanced imaging. Nevertheless, it has been recently pointed out that MRI evaluation of SVZ involvement may not fully capture the original pathology [48,49]. Adeberg et al. demonstrated that machine learning with DNA methylome-assisted classification is a more accurate method than MRI assessment of the SVZ for classifying poor prognosis groups [48]. Zhao et al. proposed an integrated approach involving T2-weighted/FLAIR imaging that can provide an improved prognostic ability [49]. There is no consensus on these methods yet, and we have not evaluated them in our cohort.

In addition to SVZ involvement, previous studies have investigated ventricular opening as a risk factor for non-local recurrence. Chan et al. proposed that ventricular opening may pose a risk of cerebrospinal fluid (CSF) dissemination and should therefore be avoided [18]. However, our data did not indicate a correlation between ventricular opening and non-local recurrence in patients with SVZ involvement, and the meta-analysis yielded a similar conclusion. These results indicate that ventricular opening does not increase the risk of non-local recurrence. Cofano et al. also showed that ventricular opening in high-grade gliomas does not carry an elevated risk of dissemination, hydrocephalus, or CSF leakage [50]. The removal of a tumor that invades the ventricular wall results in the opening of the ventricle. For cases where tumors have invaded the ventricular wall, there is an extremely high possibility that the tumor will infiltrate the ventricular wall or disseminate via the cerebrospinal fluid, as previously reported. In other words, ventricular opening does not lead to dissemination; however, dissemination is more likely when the ventricular wall is opened by removing the tumor. For cases where the tumor is in contact with the ventricular wall on MRI but has not invaded the ventricular wall, it is possible to remove the tumor while looking at the ventricular wall, and there is no need to forcefully open the ventricular wall. However, we should not be deterred from entering the ventricles to achieve a more complete tumor resection for cases where the tumors have invaded the ventricular wall.

Previous studies have yielded conflicting results regarding *MGMT* methylation as a risk factor for non-local recurrence, demonstrating significant heterogeneity [5,11,13,14,17]. The findings of a previous meta-analysis suggested that *MGMT* methylation is associated with non-local recurrence. *MGMT* promoter methylation is a prognostic factor in patients treated with chemoradiotherapy using TMZ [51]. However, the molecular basis of non-local recurrence remains unknown. One of the reasons is that patients with *MGMT* promoter methylation survive longer; therefore, non-local recurrence is often observed [13]. Recent studies have indicated that it may not be correlated with non-local recurrence [32]. In the treatment of GBM, BEV may be used as initial therapy, at recurrence, or at the end of life, and its timing varies. Therefore, ascertaining the relationship between BEV use and non-local recurrence is challenging. Postoperative ischemia has also been identified as a risk factor for non-local recurrence [33,34]. It has been proposed that the postoperative high infarct volume may initiate hypoxia-mediated aggressive tumor growth, resulting in multifocal and diffuse recurrence patterns and impaired survival [33].

This study was limited by its retrospective observational design. In addition to the lack of uniformity in the terminology and imaging definition of non-local recurrence, there may be inconsistent treatment modalities for GBM recurrence, which may be supported by the heterogeneity of the studies in the meta-analysis. We have not been able to determine the cause of these heterogeneities, and further investigation is needed to reach the ultimate conclusions. Therefore, the results obtained need to be validated in a larger cohort or multicenter prospective study.

## 5. Conclusions

SVZ involvement is associated with non-local recurrence of *IDH* wild-type GBM, and contact of the tumor with the trigone of the lateral ventricle is associated with subependymal dissemination. Ventricular opening does not increase the risk of non-local recurrence, and we should not be deterred from ventricular opening to achieve complete tumor resection.

## Figures and Tables

**Table 1:** Clinical characteristics of *IDH* wild-type GBM with a single lesion.

Characteristics (N = 104)		
Age, years, median, (IQR)	61.5	(50–72)
Male, (%)	66	(63.5)
KPS on admission, median, (IQR)	80	(70–90)
*MGMT* promoter methylation, (%)	40	(38.5)
*TERT* promoter mutation		
C228T mutation	52	(50.0)
C250T mutation	17	(16.3)
wildtype	35	(33.7)
Tumor location, (%)		
Frontal lobe	37	(35.6)
Parietal lobe	31	(29.8)
Temporal lobe	18	(17.3)
Multilobular	7	(6.7)
Infratentorial	4	(3.8)
Others	7	(6.7)
Laterality, (%)		
Left	54	(51.9)
Right	46	(44.2)
Midline	4	(3.8)
Maximum tumor size, mm, median, (IQR)	39.5	(28.2–54.0)
SVZ involvement, (%)	57	(54.8)
Initial treatment, (%)		
RT + TMZ	45	(43.3)
RT + TMZ + BEV	20	(19.2)
RT + TMZ + Gliadel wafer	6	(5.8)
Others	13	(12.5)
Ventricular opening, (%)	30	(28.8)
Extent of resection, (%)		
GTR	37	(35.6)
STR	21	(20.2)
PR	29	(27.9)
Biopsy	17	(16.3)
Postoperative ischemia, (%)	24	(23.1)
OS, months, median (95% CI)	32.1	(26.3–37.4)
PFS, months, median (95% CI)	9.6	(8.0–12.8)
Non-local recurrence free survival, median (95% CI)	41.6	(28.9–61.3)
Type of non-local recurrence, (%)		
Distant recurrence	13	(12.5)
Dissemination	25	(24.0)
Follow-up period, months, median (IQR)	24.8	(13.6–39.9)

IQR, interquartile range; KPS, Karnofsky performance status; MGMT, O-6-methylguanine-DNA-methyltransferase; TERT, telomerase reverse transcriptase; SVZ, subventricular zone; RT, radiation therapy; TMZ, temozolomide; BEV, bevacizumab; GTR, gross total resection; STR, subtotal resection; PR, partial resection; OS, overall survival; PFS, progression-free survival; CI, confidence interval.

**Table 2:** Risk factors of non-local recurrence of *IDH* wild-type GBM with a single lesion.

Factor	Univariate Analysis		Multivariate Analysis	
	Hazard Ratio (95% CI)	*p*-Value	Hazard Ratio (95% CI)	*p*-Value
Age (≥65 years)	1.527 (0.795–2.934)	0.204	NE	
Male	1.339 (0.671–2.673)	0.408	NE	
KPS on admission (≥80)	1.930 (0.995–3.744)	0.052	0.713 (0.298–1.705)	0.447
MGMT promoter methylation	0.613 (0.310–1.210)	0.159	0.520 (0.233–1.162)	0.111
*TERT* promoter mutation	0.774 (0.400–1.498)	0.447	NE	
Frontal location	0.649 (0.331–1.273)	0.209	NE	
Parietal location	1.005 (0.470–2.148)	0.990	NE	
Temporal location	1.043 (0.513–2.123)	0.907	NE	
Maximum tumor size (≥40 mm)	0.789 (0.415–1.498)	0.468	NE	
SVZ involvement	2.088 (1.076–4.048)	0.029 *	1.403 (0.576–3.422)	0.456
Ventricular opening	2.539 (1.278–5.043)	0.008 *	1.960 (0.822–4.672)	0.129
Extent of resection (GTR/STR)	1.010 (0.514–1.984)	0.977	NE	
Postoperative ischemia	1.824 (0.905–3.676)	0.093	1.118 (0.491–2.546)	0.791

NE: not entered into this model, * *p* < 0.05.

**Table 3:** SVZ involvement sites of lateral ventricle and types of non-local recurrence.

	Distant Recurrence	Subependymal Dissemination	Subarachnoidal Dissemination	Spinal Dissemination
Anterior horn	5 (33.3%)	2 (13.3%)	2 (13.3%)	1 (6.7%)
Body	0	0	0	0
Trigone	2 (7.6%)	9 (35.6%)	0	0
Inferior horn	2 (40.0%)	0	0	0
Others	1 (20.0%)	0	1 (20.0%)	0

**Table 4:** Summary of the included studies.

Author, Year	Sample Size	Incidence of Non-Local Recurrence	Observations
Liu, 2023 [11]	66	15.2%	SVZ involvement is an independent risk factor for non-local recurrence.
Yoo, 2021 [29]	358	29.5%	The patterns of recurrence are associated with the extent of resection; supratotal resection, gross-total resection, and subtotal resection.
Young, 2021 [12]	200	25.0%	There is no increase in leptomeningeal spread or distant parenchymal recurrence in patients with ventricular opening.
Jiang, 2020 [13]	247	24.7%	Being male, SVZ involvement, and *MGMT* promoter methylation are risk factors for non-local progression.
Yamaki, 2020 [14]	167	21.0%	Distant recurrence is the most frequent in the SVZ-negative and cortex-positive groups, and high CD133 expression is associated with distant recurrence.
Mistry, 2019 [15]	232	≥33.6%	SVZ contact is associated with leptomeningeal dissemination, and ventricular opening is not associated with non-local recurrence.
Kim, 2019 [30]	82	23.5%	Distant recurrence is associated with a longer time to progression.
Jungk, 2019 [16]	285	26.2%	Distant recurrence is more frequent in cortex-positive GBM.
Syed, 2018 [31]	265	34.7%	Local vs. distant brain recurrences occur similarly in both unifocal and multifocal GBM.
Schaub, 2018 [32]	142	15.5%	BEV/irinotecan (IRI) is not associated with increased rates of multifocal, distant, or highly invasive tumors at the time of recurrence.
Bette, 2018 [33]	129	44.2%	Postoperative infarct volume is associated with multifocal and diffuse recurrence patterns and impaired survival.
Rapp, 2017 [17]	97	20.6%	There is no correlation between *MGMT* methylation status and the recurrence pattern.
Chan, 2016 [18]	36	38.9%	Ventricular opening and *MGMT* methylation are associated with the development of CSF dissemination.
Adeberg, 2016 [19]	311	≥18.0%	Distant, contralateral recurrence is not influenced by ventricle opening.
Thiepold, 2015 [34]	245	27.2%	Perioperative cerebral ischemia is associated with distant or diffuse recurrence.
Adeberg, 2014 [20]	607	26.9%	SVZ involvement is associated with decreased survival and a higher risk of multifocal or distant progression.
Kimura, 2013 [21]	49	55.1%	SVZ involvement does not predict the pattern of tumor recurrence and/or extension.
Bloch, 2013 [35]	71	16.9%	The risk of dissemination does not increase due to the use of BEV.
Minniti, 2010 [22]	105	13.3%	*MGMT* methylation is associated with non-local recurrence.
Brandes, 2009 [5]	95	21.5%	*MGMT* methylation is associated with non-local recurrence.
Wick, 2008 [23]	63	20.0%	*MGMT* methylation is not associated with non-local recurrence.
Present study	104	36.5%	SVZ is associated with distant recurrence, and GBM in contact with the trigone of the lateral ventricle is associated with subependymal dissemination.

## Data Availability

The data are not publicly available due to privacy restrictions.
